# Lung Transplantation in Controlled Donation after Circulatory-Determination-of-Death Using Normothermic Abdominal Perfusion

**DOI:** 10.3389/ti.2024.12659

**Published:** 2024-05-01

**Authors:** Paula Moreno, Javier González-García, Eloísa Ruíz-López, Antonio Alvarez

**Affiliations:** ^1^ Thoracic Surgery and Lung Transplantation Unit, University Hospital Reina Sofía, Córdoba, Spain; ^2^ Group for the Study of Thoracic Neoplasms and Lung Transplantation, IMIBIC (Instituto Maimónides de Investigación Biomédica de Córdoba), University of Córdoba, Córdoba, Spain

**Keywords:** lung transplantation, normothermic regional perfusion, DCD donors, lung procurement, donation after circulatory determination-of-death

## Abstract

The main limitation to increased rates of lung transplantation (LT) continues to be the availability of suitable donors. At present, the largest source of lung allografts is still donation after the neurologic determination of death (brain-death donors, DBD). However, only 20% of these donors provide acceptable lung allografts for transplantation. One of the proposed strategies to increase the lung donor pool is the use of donors after circulatory-determination-of-death (DCD), which has the potential to significantly alleviate the shortage of transplantable lungs. According to the Maastricht classification, there are five types of DCD donors. The first two categories are uncontrolled DCD donors (uDCD); the other three are controlled DCD donors (cDCD). Clinical experience with uncontrolled DCD donors is scarce and remains limited to small case series. Controlled DCD donation, meanwhile, is the most accepted type of DCD donation for lungs. Although the DCD donor pool has significantly increased, it is still underutilized worldwide. To achieve a high retrieval rate, experience with DCD donation, adequate management of the potential DCD donor at the intensive care unit (ICU), and expertise in combined organ procurement are critical. This review presents a concise update of lung donation after circulatory-determination-of-death and includes a step-by-step protocol of lung procurement using abdominal normothermic regional perfusion.

## Introduction

Lung transplantation (LT) has become a viable life-saving therapy for patients with a variety of end-stage lung diseases. Donation after neurologic determination of death (brain death donors, DBD) remains the main source of lungs for transplantation. However, the persistent scarcity of suitable lung donors remains a major limiting factor to the number of transplants performed [1]. Among the available multiorgan donors, only 20%–25% are typically acceptable for lung donation, as the lung is particularly vulnerable to injury after brain death. To overcome organ shortage, different strategies have been applied over time, including the liberalization of standard criteria for lung donation [2], lobar lung transplantation [3], the use of donors after circulatory death (DCD donors) [4] and *ex-vivo* lung perfusion [5].

This article focuses on the expansion of DCD donors in lung transplantation, including the latest findings on the topic. We also describe our technique of combined lungs and abdominal organs procurement using normothermic abdominal perfusion step-by-step.

## Background for Donation after Circulatory Death in Lung Transplantation

In 1963, James Hardy performed the world’s first human lung transplant procedure. The patient had a squamous cell carcinoma on his left lung and recurrent episodes of pneumonia. He was a prisoner sentenced to death due to murder, which was commuted to life in prison in exchange for undergoing a lung transplant. No details on ischemic time or lung preservation were provided. The recipient survived for 18 days but finally succumbed to renal failure and “malnutrition” [6]. Just a few days later, George Magovern and Adolph Yates reported the second human lung transplant at the University Hospital in Pittsburgh [7]. The patient survived for only 1 week. It was not until 1971 that the first medium-term successful human lung transplantation was performed by Fritz Derom in Belgium [8]. Both the first world’s lung transplant and the first medium-term successful human lung transplants utilized DCD donors. Over the next two decades, approximately 38 lung, lobe, or heart-lung transplant procedures were attempted, with no long-term success [9, 10]. All used DCD donors, as formal criteria for brain death had not yet been established. When the Harvard criteria for brain death were accepted [11], DBD became the standard method for organ donation, with DCD donation being abandoned.

DCD donation was then reintroduced by Thomas M. Egan in 1991, following a series of canine experiments demonstrating its feasibility [12]. In 1995, Robert Love reported the first controlled DCD lung transplant with success. Loves’s group performed a left single lung re-transplantation in a patient on ECMO for severe primary graft dysfunction (PGD) [13]. An international workshop organized in 1995 led to the Maastricht classification of DCD donors [14], to categorize DCD donors based on the duration of warm ischemia. This classification has since been updated [15–17].

Steen and others reported a successful right single LT from a uDCD donor after failed resuscitation in 2001 [18]. The authors preserved the lungs by topical cooling inside the body of the potential donor, while consent for donation was obtained from the next of kin, and *ex-vivo* evaluation of lung function (EVLP) was done. EVLP is crucial when considering uDCD for LT, as premortem functional evaluation is not possible. Worldwide experience with uDCD donors is still limited to small case series, as logistics are complex.

## Definitions and Categories of Donation after Circulatory Death

### Maastricht Classification

The Maastricht classification organizes DCD into five categories. Categories I (dead on arrival) and II (unsuccessful resuscitation) are considered “uncontrolled” donors (uDCD), whereas categories III (awaiting cardiac arrest), IV (unexpected cardiac arrest in a brain-dead donor), and V (euthanasia) comprise “controlled” DCD (cDCD) (Table 1) [17]. In the uncontrolled DCD scenario, a patient suffers from sudden death, so cardiopulmonary resuscitation maneuvers are initiated and continued during transport to hospital. In controlled DCD, a patient with catastrophic brain injury for whom supportive care is thought to be futile is subjected to withdrawal of life-sustaining therapies (WLST) in a planned way. Nowadays, controlled DCD donation is the most used DCD type used for transplantation.

**TABLE 1:** Modified Maastricht classification of donation after circulatory death [15].

Category		Definition	Subclassification	
Uncontrolled	I	Found dead	Ia	Out-of-hospital
			Ib	In hospital
	II	Witnessed cardiac arrest	IIa	Out-of-hospital
			IIb	In hospital
Controlled	III	Planned WLST	IIIa	In ICU
			IIIb	In OR
	IV	Cardiac arrest while brain death prior to organ recovery	IVa	Unexpected in ICU
			IVb	Expected in OR/ICU
	V	Medically assisted death/euthanasia	Va	Out-of-OR
			Vb	In OR

ICU, Intensive Care Unit; OR, Operating Room; WLST, Withdrawal of life-sustaining therapies.

### Warm Ischemia

Organs retrieved from DCD donors are vulnerable to warm ischemic injury, especially the heart and the liver. However, the lung is unique among solid organs that are transplanted, as the alveoli remains filled with oxygen despite not being perfused. Nevertheless, this is an important difference to DBD, as in DCD an additional warm ischemia interval exists. Worldwide, there is no consensus regarding the definition of warm ischemic time in DCD donors. Whereas WIT can be generally described as the time between the withdrawal of life-sustaining therapies (WLST) and the initiation of organ preservation (*total* WIT), the critical period starts when significant hypoperfusion occurs. This defines the *functional WIT* and corresponds to a drop in systolic blood pressure below 50 mmHg. In Spain, the cut-off of systolic arterial pressure is slightly higher, at 60 mmHg. Some additional terms and definitions have been also proposed:

-**Relative WIT:** From WLST to significant organ hypoperfusion (mean arterial pressure <50 mmHg). **Absolute or functional WIT** is the time between significant organ hypoperfusion (mean arterial pressure <50 mmHg) and initiation of organ preservation.
**Acirculatory WIT:** From cardiac arrest to cold flush. **Agonal phase**: from WLST to circulatory arrest. **No-touch period**: from circulatory arrest to death declaration. **Warm to cold interval**: from death declaration to cold preservation.

**FIGURE 1:**
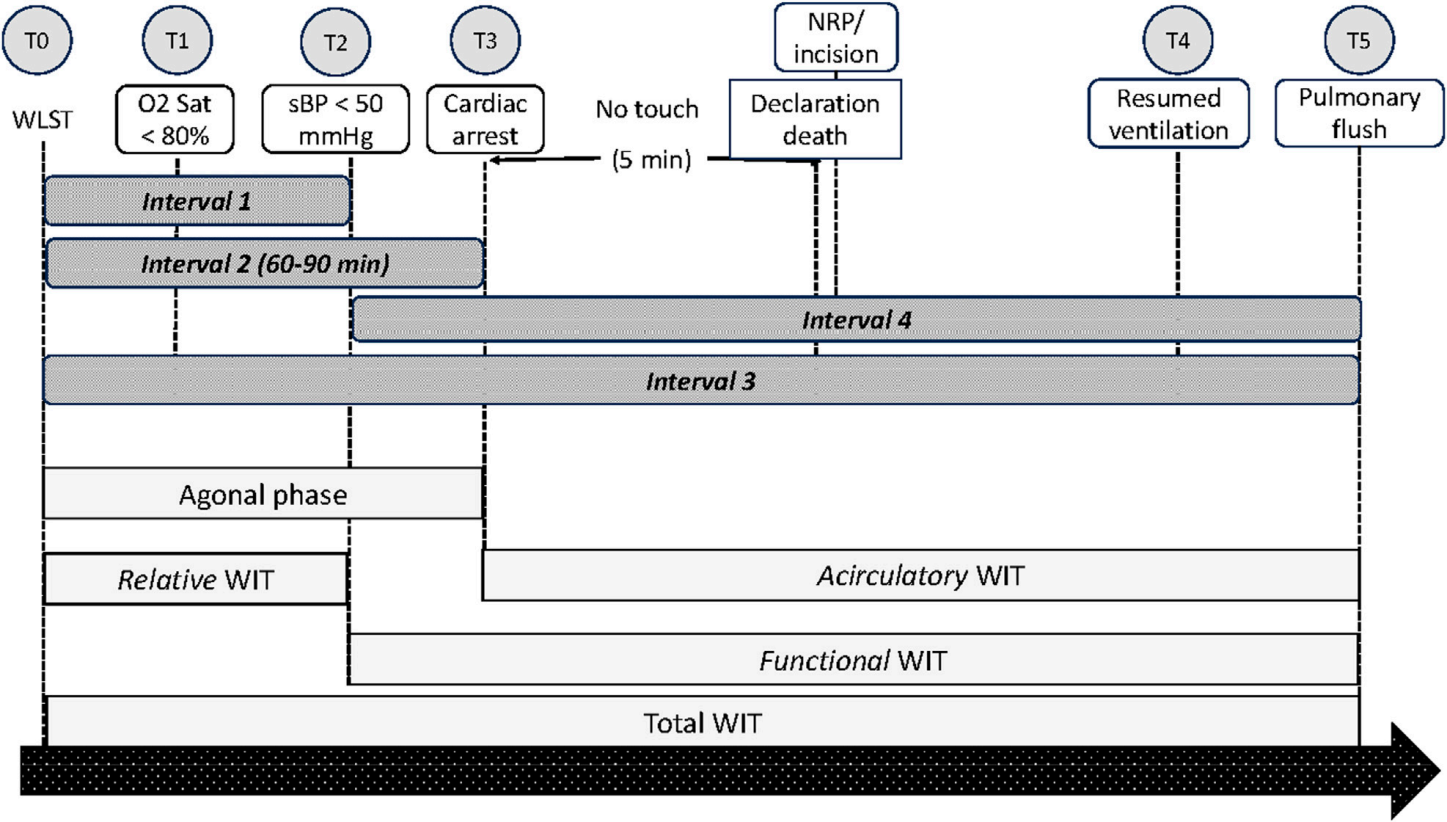
Schematic presentation of time points and intervals as suggested by the ISHLT DCD Working Group.

The International Society for Heart and Lung transplantation (ISHLT) DCD Working Group recommended different time points and intervals for lung donation, as depicted in Table 2; Figure 1 [19]:

-T0: withdrawal of life-sustaining therapies or euthanasia
-T1: oxygen saturation <80%
-T2: systolic blood pressure <50 mmHg
-T3: cessation of cardiac output/asystole
-T4: resumed lung inflation/ventilation
-T5: start of pulmonary flush

**TABLE 2:** Major time-points suggested by ISHLT DCD Working Group [19].

Time Point	Description
T0	Withdrawal of life-sustaining therapies or euthanasia
T1	Oxygen saturation <80%
T2	Systolic blood pressure <50 mmHg
T3	Cessation of cardiac output/asystole
T4	Resumed lung inflation/ventilation
T5	Start of pulmonary flush

ISHLT, International Society for Heart and Lung Transplantation; DCD, donation after cardiac death.

Interval 1 is the time from the withdrawal of life-sustaining therapy (WLST) to hypotension with systolic blood pressure <50 mmHg (T0 to T2); Interval 2 is the time from WLST to cessation of cardiac output/asystole (T0 to T3); Interval 3 is the time from WLST to start of pulmonary flush; and Interval 4 is the time from systolic blood pressure <50 mmHg to start of pulmonary flush (T2 to T5).

According to the ISHLT DCD Registry Report, no differences in one-year survival after LT were observed irrespective of the duration of Intervals 1, 2, or 3 [19].

The acceptable upper limit of WIT for DCD LT is still debatable [20]. During the agonal phase, progressive hypoxemia and hypoperfusion occur before cardiac arrest. This interval varies among donors and may result in organ injury. It has been suggested that the most important parameters associated with organ damage are oxygen saturation below 85% and systemic arterial pressure below 50 mmHg [21]. On the contrary, in a multicenter ISHLT DCD Registry analysis, the authors did not find an association between the duration of the agonal phase or functional WIT up to 60 min and early survival after LT [22]. In this report, 84.5% of 465 DCD donors reached asystole in 30 min and 96.5% reached it in >60 min after WLST. Furthermore, the Toronto group has reported good outcomes using DCD donors taking more than 120 min from WLST to cardiac arrest [23]. Nevertheless, most centers agree on 60–90 min of WIT [22].

An important difference between DCD and DBD is the possibility of autoresuscitation in DCD donors when spontaneous resumption of cardiopulmonary activity after circulatory arrest occurs. Thus, a “no-touch” or observation period has been established. This timeframe is debatable and depends on legal and ethical criteria. Many countries accept no-touch periods between 2 to 5 min, although this is variable and may extend up to 20 min, such as in Italy [24].

## Donor Selection Criteria

The DCD donor selection criteria are the same as for DBD. It is of paramount importance to adequately select DCD donors to reduce the risk of primary graft dysfunction (PGD) after LT. Assessment of lung function in DCD is performed by bronchoscopy, chest x rays, arterial blood gases, and inspection. Potential cDCD donors are critically ill patients with irreversible brain injury or end-stage musculoskeletal disorders who are expected to have circulatory arrest after WLST [25]. Ideally, a cDCD donor should arrest in less than 60 min. Different models have been developed to predict the likelihood of progression to asystole after WLST, like the University of Wisconsin donation after circulatory death evaluation tool [26]. However, they are not 100% accurate, as some donors will not develop cardiac arrest within 120 min, leading to aborted procedures. To date, no reliable models exist to estimate time to circulatory death in DCD donors, and the accuracy of the available ones is modest [26–29].

The rate of aborted procedures (so-called dry-runs) is variable, ranging from 40% [30] to 1% of aborted procedures in Belgium [31]. Possible explanations to this wide variability in the rate of dry-runs are differences in existing legal frameworks, ethical barriers, and lack of technical expertise or required logistics. Moreover, proficiency of the donor coordinator in identifying and selecting a potential DCD donor is associated with low rates of dry-runs [32].

In addition to aborted procedures, DCD donation may be considered as expensive, as it has been reported that the cost per organ from a DCD is 60% higher compared to DBD [33]. Transplant groups may be reluctant to send a team to a donor hospital for fear the donor does not progress into cardiac arrest in an acceptable time, considering the high costs of travel, personnel, and the surgical procedure, added to complex logistics. Further, DCD procurement requires specialized training, especially when joined thoracic and abdominal procurement is planned. In addition, the exceeded costs in DCD organ procurement can be attributable to the higher rate of dry-runs in the earlier experience, mainly secondary to transportation and surgeon fees for declined organs [34] It is expected that, with the expansion of DCD programs, the costs will be reduced to almost those of DBD donation. Likewise, DCD organ donation is cost-effective, as the costs of maintaining the potential candidates on the waiting list expecting to receive a suitable DBD organ donor are probably higher than the benefits on survival and quality of life after receiving an organ from a DCD donor.

Another issue of concern is organ retrieval rate from DCD donors. Organ yield after DCD is lower than DBD. Although the activity has increased significantly worldwide, DCD donors are still underutilized, representing only 2% of LT in the United States and 5% in Europe. This contrasts with higher rates in Australia (28%), the Netherlands (40%), England (25%), and Canada 32% [35–37].

A recent study using data from the United Network for Organ Sharing (UNOS) revealed that, from 30,916 lungs, only 3.8% (1158) were used for transplantation between 2005 and 2009, and nearly 73% were discarded, mainly due to poor lung function [38]. PaO2/IO2 ratio below 250, smoking history, or clinical infection with a blood source were identified as predictors of non-use.

Protocols for DCD donation may differ depending on several factors, including the location of WLST, premortem interventions, withdrawal of tracheal tube, duration of the no-touch period and WIT, and the possibility of *ex-vivo* lung perfusion (EVLP). The consensus document of the ISHLT covers essential aspects of the DCD donor procurement process, including NRP, *ex-vivo* evaluation, and declaration of circulatory death [39].

a) The location of WLST: Operating Room vs. Intensive Care Unit. The location of WLST has a critical impact on DCD organ recovery rate and post-transplant outcomes as, when performed in the ICUs, it lengthens WIT. Consequently, WLST is preferably performed in the operating room (OR) to minimize WIT. Alternatively, it can be done at the intensive care unit followed by transport of the donor to the OR and reintubation.
b) Premortem interventions: These include comfort therapy, pre-arrest heparinization, or premortem bronchoscopy. These practices vary among transplant centers due to ethical considerations. The administration of comfort therapy during WLST could indirectly affect the duration of the agonal period and this topic remains debatable. However, current evidence about the role of sedatives in accelerating death does not provide definitive results [40].
c) The allowed WIT: The interval from WLST to declaration of death is variable and ranges from 60 to 180 min. However, most groups accept 60 min of WIT.
d) Withdrawal of tracheal tube. Protection against aspiration by avoiding extubation and just stopping ventilation is preferred at our Institution. On the contrary, withdrawal of the tracheal tube followed by re-intubation and re-ventilation after declaration of death and the no-touch period can be performed.
d) Placement of nasogastric tube during WLST is routine practice to prevent aspiration of gastric contents and to facilitate dissection around the esophagus.
e) Time of re-ventilation: Re-ventilation is initiated after median sternotomy is performed. This time is important as the maneuver may be associated with autoresuscitation. Thus, many groups wait for at least 5 min after surgical incision, or up to 15 min after cardiac arrest, like in Australia [21].
f) The duration of the no-touch period: as stated before, most LT groups use no-touch periods that range from 2 to 5 min, although they may be as long as 20 min, such as in Italy.
g) The possibility of *ex-vivo* lung perfusion (EVLP): according to the ISHLT report published in 2015, EVLP was used in only 15% of DCD donors, reflecting that EVLP technology is not available in many LT groups. The majority of EVLP runs were reported by the Toronto group [19]. Excellent outcomes of LT from cDCD donors without EVLP have been reported [31]. On the contrary, the use of EVLP is strongly recommended by the ISHLT to evaluate uDCD donors.
h) Heparinization: In the DCD setting, heparin can be administered either pre-mortem or post-mortem. However, there are some ethical concerns, as pre-arrest heparinization is not allowed in all countries due to the possibility of accelerating death in the potential donor. Whereas Oto and others reported a 38% incidence of unexpected donor thromboembolism which was associated with increased rates of PGD after LT [41], other groups have found that delayed heparin administration after cardiac arrest or even not administering it at all are associated with good results. According to the ISHLT DCD Registry, pre-mortem heparin was given in 54% of DCD donors. Interestingly, this not correlated with adverse outcomes after LT [19]. Table 3 shows some features of the existing legal framework and invasive procedures used in controlled DCD donation in Europe.

**TABLE 3:** Available legal framework and invasive procedures used in controlled DCD donation [24].

Country	Ante-mortem medication	Ante-mortem cannulation	Location of WLST	No-touch period (min)
Austria	Yes	Yes but not practiced	OR	10
Belgium	Yes	Yes	OR	5
Czech Republic	No	No	ICU	5
France	Yes	Yes (only guidewires)	ICU	5
Ireland	No	No	OR	10
Italy	Yes	Yes (only guidewires)	ICU	20
Netherlands	No	No	ICU	5
Norway	Yes	Yes (only guidewires)	ICU	5
Spain	Yes	Yes	OR	5
Sweden	No	No	ICU	5
Switzerland	Yes	No	ICU	5
United Kingdom	No	No	ICU	5
USA	Yes	Yes	No standard practices	5
Australia	Variable	NRP not allowed	ICU	5

ICU, Intensive Care Unit; OR, Operating Room; WLST, Withdrawal of life-sustaining therapies.

## Joint Thoracic and Abdominal Organ Procurement Prom DCD Donors

Combined thoracic and abdominal organ procurements from DCD donors are standard practice worldwide. Traditionally, standard organ procurement from DCD donors has involved simultaneous super rapid recovery (SSR) of lungs and abdominal grafts, by means of cold thoracic and abdominal perfusion. This technique continues to be the procedure of choice in some countries. Advantages of SSR from DCD donors are the reduced costs, availability, reproducibility, and the familiarity with the procedure [42]. Nevertheless, SSR of organs from DCD donors require expertise and surgical skills to minimize the risks of organ injury or even graft loss secondary to surgical accidents [43].

Ideally, WLST is performed in the operating room with the potential DCD donor prepped and draped in a sterile fashion and the surgical instruments on the table, ready to be used and, therefore, shortening WIT. In addition, both the thoracic and abdominal teams are scrubbed, and all instruments required are prepared on the instrument table. A separate side table for the thoracic team is desirable, and pulmoplegia is prepared by adding 500 micrograms of Prostaglandin E1 (Alprostadil) to the first bag of Perfadex solution. If the legal framework allows for it, a single bolus of 500–100 Units of heparin sodium is administered. Alternatively, heparin can be added to flush solutions.

### Normothermic Regional Perfusion (NRP)

Whereas the outcomes of LT from DCD donors using the technique of SRR are similar or even better than from DBD, as the negative factors associated with brain death are avoided, DCD livers face higher rates of primary non-function, early graft dysfunction, and biliary complications [44]. In addition, DCD kidneys show a higher incidence of delayed graft function compared to DBD [45]. The negative results of abdominal organ transplantation from DCD donors rely on the warm ischemic damage during the hypotensive phase after WLST and, thereafter, during cold ischemia prior to organ reperfusion in the recipient. Thus, normothermic regional perfusion using extracorporeal membrane oxygenation (ECMO) has gained increased interest in recent years [46].

Normothermic regional perfusion (NRP) consists of the use of extracorporeal membrane oxygenation (ECMO) to perfuse organs at normothermia after death declaration prior to organ recovery [47]. Perfusion can be limited to the abdominal cavity (A-NRP) or both the thorax and abdomen (TA-NRP). TA-NRP has facilitated heart procurement from DCD donors, reducing warm ischemic time and allowing for *in situ* evaluation of donor graft function, and its use has expanded in the United States and Australia [48, 49].

NRP restores the flow of oxygenated blood after cardiac arrest, to reverse warm ischemic injury of abdominal organs following the determination of death and before organ recovery. NRP enables assessment of organ function, as opposed to *in situ* cooling and rapid procurement [46]. Furthermore, the use of A-NRP is associated with a lower incidence of ischemic cholangiopathy, liver graft dysfunction, and delayed kidney graft function [44, 50]. Santander’s group in Spain reported the largest single center study reporting on combined lung and liver procurement from DCD donors using normothermic abdominal perfusion. In this study, the authors included 60 lung transplants from cDCD and compared the results with 209 LT from DBD donors [51]. In Europe, NRP is applied for DCD organ procurement in France, Italy, Spain, the UK, Belgium, the Netherlands, Norway, and Switzerland [24]. In Spain NRP is used routinely, whereas it is mandated for liver procurement in France and Italy.

### DCD Donor Cannulation

Cannulation of the donor for NRP can be performed either before or after WLST, depending on the existing national legal framework. In Spain, pre-mortem cannulation and heparinization are allowed to reduce WIT [52]. In the Spanish setting, appropriate consent is obtained from the next of kin for any pre-mortem interventions, including heparinization and femoral cannulation for NRP. Antemortem heparinization is allowed in Belgium, France, Norway, Spain, and Italy. Whereas antemortem cannulation is allowed only in Spain and Belgium, in other countries like France, Italy, and Norway antemortem vessel localization by guidewires can be performed. On the contrary, no antemortem interventions are allowed in the UK or the Netherlands [53].

#### Ante-Mortem Vessel Cannulation

Peripheral femoral vessel cannulation is the preferred method for ante-mortem NRP. In some countries, the femoral vessels are identified prior to WLST but cannulation is performed after the declaration of death [24]. Preparation, management of the potential cDCD donor, and surgical technique have been reported previously [47, 52]. The process starts by verifying that the left radial artery is already catheterized. The next step is cannulation of the femoral artery (15-Fr to 19-Fr) and vein (18-Fr to 21-Fr), either surgically or using the Seldinger technique, under proper sedation and analgesia. After guidewires are placed, 500–600 units/Kg of heparin are administered prior to vessel cannulation. The cannulas are de-aeriated and connected to the ECMO circuit, with the heater/cooler at 37°C but the pump off. Through the contralateral groin, an aortic occlusion balloon is placed and advanced until empty and reaching the supraceliac aorta, to ensure the thoracic aorta is adequately blocked during A-NRP to avoid the possibility of autoresuscitation [54]. Alternatively, the descending thoracic aorta can be directly occluded above the diaphragm by a vascular clamp immediately after median sternotomy, to ensure the heart and the brain are not perfused during A-NRP.

Two arterial lines (left radial artery and another from the femoral artery cannula) are monitored during A-NRP to ensure the thoracic aorta is appropriately blocked [52, 54]. For this purpose, just before WLST the aortic occlusion balloon is filled for 4 s and its position checked by X-ray or fluoroscopy, after which the balloon is immediately emptied. The tip of the catheter should be 6-7 cm above the xiphoidal process, between the left subclavian artery and the celiac trunk. The thoracic surgeon performs a fiberoptic bronchoscopy as in DBD donors, if premortem interventions are allowed. Otherwise, this can be performed after the declaration of death, while one of the thoracic surgeons performs median sternotomy.

**FIGURE 2:**
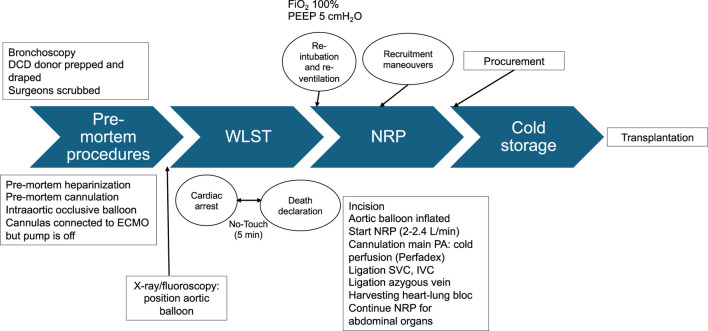
Step by step schematic presentation of DCD organ procurement under NRP. WLST, Withdrawal life sustaining therapies; NRP, normothermic regional perfusion. ECMO, extracorporeal membrane oxygenation; PA, pulmonary artery; SVC, superior vena cava; IVC, inferior vena cava. FiO2, Fraction of inspired oxygen; PEEP, Positive end expiratory pressure.

When the preparation of the potential DCD donor has finished, the surgical field is prepped, and the team proceeds with WLST including donor extubation, according to local protocols. Some groups prefer to leave the tracheal tube on site, instead of removing it, to reduce the risk of aspiration. Separate trolleys for thoracic and abdominal teams are set up with the necessary instruments and flush solutions. The surgical teams are scrubbed in an adjacent OR. After 5 min of the no-touch period, death is certified by the permanent cessation of circulation (pulseless arterial line, continuous apnea, unresponsiveness). At this point, the aortic occlusion balloon is filled, and ECMO is started, and flow is progressively increased until reaching 2–2.4 L/min. At this time, organ procurement can proceed. The thoracic and abdominal teams enter the OR and perform median sternotomy and laparotomy, respectively. The donor is then re-intubated and mechanical ventilation resumed at FiO_2_ 100%, PEEP of 5 cm H_2_O and a tidal volume of 6–8 mL/kg. For lung perfusion, the pericardium is opened, and the main PA cannulated. 50–60 mL/kg of Perfadex primed with Prostaglandin E1 is delivered and the left atrial appendage is opened for drainage. Meanwhile, both pleurae are opened widely and cold Perfadex or cold saline are used for moderate topical cooling of both lungs. Macroscopic inspection of the lung grafts is performed to anticipate suitability (Figure 2).

To prevent ascending flow secondary to mispositioning or displacement of the aortic occlusive balloon, the descending aorta above the balloon can be isolated to allow the placement of an aortic clamp. When the pulmonary flush is finished, the ascending aorta is isolated and transected preferably using staplers rather than placing ligatures, as well as both superior and inferior vena cava. This technique shortens lung procurement, minimizing WIT, more so than placing ligatures or vascular clamps. Moreover, meticulous hemostasis is better achieved, which is of paramount importance for adequate functioning of the A-NRP. Before transecting the inferior vein cava, the position of the return cannula within the right atrium is checked, and the tip is withdrawn below the diaphragm. About 1–1.5 L of fluid are administered intravenously to avoid a decrease in blood flow to the ECMO circuit. A minimum of 30 min and a maximum of 4 h are stipulated for NRP, but 90–120 min are routine practice nowadays [52, 55]. Nevertheless, the optimal duration of NRP remains debatable.

Once lung cold preservation has finished, the heart-lung bloc is removed. The heart is removed first in a standard fashion, followed by the double-lung graft. Care should be taken when dissecting the azygous vein, which is transected using staplers. Once the heart-lung bloc is removed, the thoracic cavity is checked for bleeding. Once on the back table, retrograde flush perfusion through each pulmonary vein is made until the effluent runs clear from the PA as with DBD donors. Finally, the lung bloc is triple-bagged stored in cold Perfadex and transported to the transplant center.

Target parameters of NRP are flow of 2–2.4 L/min, pH 7.35–7.45, temperature of 37°C, and hematocrit >25%. Blood samples from the ECMO circuit are obtained immediately after starting A-NRP and every 30 min, monitoring liver enzymes, lactate levels, urea, electrolytes, and blood gases. For liver graft acceptance, alanine transaminase or aspartate transaminase levels at 30 or 60 min after the initiation of NRP should be <4 times the normal values, together with normal macroscopic appearance and declining lactate levels.

#### Post-Mortem Vessel Cannulation

Post-mortem cannulation can be performed either in the abdomen or in the common femoral vessels.

The technique of post-mortem cannulation in NRP has been reported previously [56, 57]. After death declaration, a rapid laparotomy is performed with aortic and inferior vena cava cannulation (or iliac vein and artery). The descending thoracic aorta is occluded either by a vascular clamp or by an occluding intra-aortic balloon. Of note, pre-mortem heparin administration is prohibited in the UK. In case of combined lung and liver procurement, the thoracic team performs a rapid median sternotomy for lung harvesting, while the abdominal team performs laparotomy and vessel cannulation for A-NRP [58]. In this case, fiberoptic bronchoscopy is performed once death has been declared, while one thoracic surgeon opens the thorax. Post-mortem cannulation for A-NRP increases functional WIT by 10–23 min [57].

#### Preparation and Priming of ECMO

A basic ECMO circuit for NRP consists of a centrifugal pump, a membrane oxygenator, the heat exchanger, a central unit controller, tubing and cannulas. For priming, 1.5 L of crystalloid solution and 30 mg of 1% heparin sodium are added to the perfusate to maintain activated clotting time between 180 and 200 s. In addition, bicarbonate is usually needed to maintain pH between 7.35 and 7.45, as donor acidosis is frequent. Packed red blood cells are also added if hemoglobin levels fall below 8 g/dL.

### Problems and Solutions During A-NRP

Main problems during A-NRP for organ procurement from DCD donors are reaching very low flows while on NRP, which can lead to organ loss, and the possibility of restoration of perfusion to the heart or the brain, the so-called autoresuscitation of the donor. Sudden loss of venous return is usually observed during lung procurement, secondary to bleeding or due to the collapse of the inferior vena cava. Thus, meticulous hemostasis and ligation of the azygous vein is of paramount importance. Moreover, care should be taken while dissecting the inferior vein cava to avoid compromising venous return.

### Autoresuscitation

One of the major concerns is the possibility of autoresuscitation of the donor during NRP. A correctly placed occlusive aortic balloon during NRP should translate into an absence of pulsatile wave form from the left radial artery and into a continuous, non-pulsatile pressure from the femoral arterial line. On occasion, a non-pulsatile wave form from the left radial artery can be observed after A-NRP has started, due to incorrect positioning or inadequate filling of the aortic balloon. In this case, ECMO flow is immediately stopped, the position of the aortic balloon is checked, and an additional 5 min of the no-touch period are observed before A-NRP is re-started [54].

### Thoraco-Abdominal NRP

*In situ* thoraco-abdominal regional perfusion (TA-NRP) has emerged recently as an alternative method to direct heart procurement (DP) with *ex-situ* machine perfusion. The technique was used for the first time in the UK [59] to allow heart transplantation from DCD donors and has increased dramatically in the USA [60]. Following declaration of death and 5 min of the no-touch period, a rapid median sternotomy is performed, the pericardium is opened, and the brachiocephalic trunk, left carotid artery, and left subclavian artery are either clamped or divided to avoid brain perfusion. TA-NRP is initiated once the right atrium and ascending aorta have been cannulated and the intra-aortic occlusive balloon deflated. Either standard cardiopulmonary bypass (CPB) or veno-arterial ECMO can be used for extracorporeal perfusion. TA-NRP is gradually weaned after 30–60 min once the heart returns to sinus rhythm. Validity of the heart is clinically assessed, as it should be able to perfuse thoracic and abdominal organs. Swan-Ganz catheter, trans-esophageal echocardiography, and visual inspection and palpation are used for that purpose.

Simultaneously, the donor is re-intubated and mechanical ventilation resumed. Targeted parameters during TA-NRP are medium systolic arterial pressure >50 mmHg, flows >2.5 L/min/m^2^, and normothermia. According to the Papworth group, median duration of TA-NRP is 45 min (27–190 min) and the donor heart is routinely placed on the OCS Heart Device for transportation [61].

The use of TA-NRP raises some ethical issues, as the permanence principle of death might be violated if the brain is perfused while on NRP. Leaving the distal ends of the aortic vessels vented to atmosphere or cannulating each of them and connecting them to the venous return, as well as inserting a cannula in the ascending aorta to ensure absence of flow to the brain [62], are some of the suggested measures in this regard. Measurement of mean intracranial pressures at the circle of Willis in DCD donors during TA-NRP has clearly demonstrated that these specific measures are effective in avoiding perfusion to the brain [63].

## Outcomes

NRP in DCD is a technically demanding procedure with more complexity and costs than DBD. Early experience in LT from cDCD using abdominal normothermic perfusion for combined lung and liver procurement came from single case reports by the Newcastle and Birmingham groups in the UK [58]. Later, Miñambres and others from Santander proposed a modified technique including pre-mortem interventions, as the legal framework exists in Spain [64]. In Spain, abdominal *in-situ* NRP with super rapid recovery of the lungs has become the standard technique of DCD organ procurement [65]. Looking at the last report of the Spanish Transplant Organization, 36% of LT performed in 2022 had cDCD donors [66]. The Puerta de Hierro group in Madrid reported for the first time the results of LT from DCD donors under A-NRP compared to classic SRR [67]. The authors did not find any difference in the incidence of PGD, hospital mortality, or one-year survival. Moreover, the complexity of the procedure did not impact negatively on the procurement rate of abdominal organs. Recently, a multicenter study including all LT centers in Spain has analyzed the outcomes of simultaneous lung and liver recovery using A-NRP compared to those of contemporary DBD donors [68]. The rates of grade 3 PDG at 72 h and lung transplant survival at 1 and 3 years were similar between recipients from DCD and DBD donors. Moreover, the incidence of liver graft dysfunction, ischemic cholangiopathy, or liver graft survival did not differ between DBD and DCD donors.

### Organ Recovery

Combined lung and liver procurement from cDCD donors using regional perfusion is more complex due to the use of ECMO and dual temperature (cold in the thorax, normothermia in the abdomen) which may have deleterious effects on the grafts.

The Santander group has reported fantastically high recovery rates for both lungs (97.4%) and livers (84.2%) [64]. The multicenter Spanish experience reported recently showed lower recovery rates for livers (78% compared to 85.5% in DBD), but similar percentages for the lungs (73.9% vs. 75.8%) [68]. A systematic review and meta-analysis or regional perfusion in DCD solid organ transplantation has reported 25%–100% organ utilization rates for the liver and 0%–60% for the lungs [69]. A French multicenter study published recently has reported a DCD lung transplantation rate of 76% [70]. From 100 controlled DCD lung grafts offered, 10 were not retrieved due to prolonged agonal phases, failure of cannulation for NRP, or poor *in-situ* evaluation. The remaining 90 were subjected to *ex-vivo* lung perfusion (EVLP) and finally 76 were accepted for transplantation. In the same period, 412 livers from DCD donors were transplanted. Combined lung and liver procurement was performed in 59 cases, and the remaining were isolated liver procurements. In combined procurements, no difference in overall survival for livers and kidneys were observed, compared to isolated abdominal organ procurement.

### Graft Quality and Organ Dysfunction

No differences on PaO2/FiO2 at ICU have been observed compared to DBD donors. What is more, A-NRP seems to decrease the rate of grade 1 PGD (4.8% vs. 7.4%) and grade 2 PGD (4.8% vs. 9.6%). On the contrary, a higher incidence of grade 3 PGD has been reported (19% vs. 7.4%) [64]. In contrast, the Spanish multicenter study has not found a higher incidence of grade 3 PGD [68]. Similarly, a single-center retrospective study of DCD lung transplantation from 2013 to 2019 comparing A-NRP with SRR has found a 21% incidence of PGD in both groups (P-1.0) [67].

### Mortality and Survival

In most DCD LT series, 30-day mortality and midterm survival rates are similar between cDCD and DBD LT. Identical results have been reported when A-NRP is used. Campo-Cañaveral De la Cruz and others from Spain have reported a 30-day mortality rate of 5.6% in DCD and 6.3% in DBD [68]. Recipient survival at 1 and 3 years for cDCD LT was 79.9% and 66.4% vs. 82% and 69.7% in DBD respectively (*p* = 0.403) in this Spanish cohort. Two-year survival was 84% in cDCD compared to 90% in DBD according to the Santander experience [64].

## Summary

Scarcity of suitable lung donors and the low usage rates of donor lungs remain major limitations to increasing the number of LT performed. Traditionally, the main source of lung allografts has been DBD donors. However, a resurgence in DCD LT has occurred to alleviate lung donor shortage. DCD LT has increased exponentially since the mid-90 s, with comparable results to DBD LT. The ISHLT has provided a consensus document for heart and lung procurement from DBD and DCD donors, standardizing the procedure, terminology, time-points, and intervals. The Maastricht classification of DCD donors has been subsequently updated to include the location of cardiac arrest and category V, consisting of euthanasia.

Recent expansion of regional normothermic perfusion for abdominal organ recovery from DCD is a growing approach in increasing the number of LT performed. Although adding complexity to the procedure of multiorgan procurement, A-NRP improves abdominal organ recovery rates and organ function.

## References

[1] Valapour M Lehr CJ Skeans MA Smith JM Uccellini K Lehman R OPTN/SRTR 2017 Annual Data Report: Lung. Am J Transpl (2019) 19(Suppl. 2):404–84. 10.1111/ajt.15279 30811892

[2] Bhorade SM Vigneswaran W McCabe MA Garrity ER. Liberalization of Donor Criteria May Expand the Donor Pool without Adverse Consequence in Lung Transplantation. J Heart Lung Transpl (2000) 19(12):1199–204. 10.1016/s1053-2498(00)00215-1 11124490

[3] Aigner C Mazhar S Jaksch P Seebacher G Taghavi S Marta G Lobar Transplantation, Split Lung Transplantation and Peripheral Segmental Resection--Reliable Procedures for Downsizing Donor Lungs. Eur J Cardiothorac Surg (2004) 25(2):179–83. 10.1016/j.ejcts.2003.11.009 14747109

[4] Dalessandro AM Hoffmann RM Knechtle SJ Eckhoff DE Love RB Kalayoglu M Controlled Non-heart-beating Donors: A Potential Source of Extrarenal Organs. Transpl Proc (1995) 27(1):707–9. 7879152

[5] Ingemansson R Eyjolfsson A Mared L Pierre L Algotsson L Ekmehag B Clinical Transplantation of Initially Rejected Donor Lungs after Reconditioning *Ex Vivo* . Ann Thorac Surg (2009) 87(1):255–60. 10.1016/j.athoracsur.2008.09.049 19101308

[6] Hardy JD Webb WR Dalton ML Jr. Walker GR Jr. Lung Homotransplantation in Man. JAMA (1963) 186:1065–74. 10.1001/jama.1963.63710120001010 14061414

[7] Magovern GJ Yates AJ. Human Homotransplantation of Left Lung: Report of a Case. Ann N Y Acad Sci (1964) 120:710–28. 10.1111/j.1749-6632.1964.tb34764.x 14235285

[8] Derom F Barbier F Ringoir S Versieck J Rolly G Berzsenyi G Ten-Month Survival after Lung Homotransplantation in Man. J Thorac Cardiovasc Surg (1971) 61(6):835–46. 10.1016/s0022-5223(19)42145-4 4932557

[9] Nelems JM Rebuck AS Cooper JD Goldberg M Halloran PF Vellend H. Human Lung Transplantation. Chest (1980) 78(4):569–73. 10.1378/chest.78.4.569 6998666

[10] Wildevuur CR Benfield JR. A Review of 23 Human Lung Transplantations by 20 Surgeons. Ann Thorac Surg (1970) 9(6):489–515. 10.1016/s0003-4975(10)65544-0 4193736

[11] A definition of irreversible coma. Report of the Ad Hoc Committee of the Harvard Medical School to Examine the Definition of Brain Death. JAMA (1968) 205(6):337–40. 5694976

[12] Egan TM Lambert CJ Jr Reddick R Ulicny KS Jr. Keagy BA Wilcox BR. A Strategy to Increase the Donor Pool: Use of Cadaver Lungs for Transplantation. Ann Thorac Surg (1991) 52(5):1113–20. 10.1016/0003-4975(91)91290-c 1953132

[13] Egan TM Haithcock BE Lobo J Mody G Love RB Requard JJ 3rd Donation after Circulatory Death Donors in Lung Transplantation. J Thorac Dis (2021) 13(11):6536–49. 10.21037/jtd-2021-13 34992833 PMC8662509

[14] Kootstra G Daemen JH Oomen AP. Categories of Non-heart-beating Donors. Transpl Proc (1995) 27(5):2893–4. 7482956

[15] Detry O Le Dinh H Noterdaeme T De Roover A Honore P Squifflet JP Categories of Donation after Cardiocirculatory Death. Transpl Proc (2012) 44(5):1189–95. 10.1016/j.transproceed.2012.05.001 22663982

[16] Evrard P, Belgian Working Group on DCD National Protocol. Belgian Modified Classification of Maastricht for Donors after Circulatory Death. Transpl Proc (2014) 46(9):3138–42. 10.1016/j.transproceed.2014.09.169 25420844

[17] Thuong M Ruiz A Evrard P Kuiper M Boffa C Akhtar MZ New Classification of Donation after Circulatory Death Donors Definitions and Terminology. Transpl Int (2016) 29(7):749–59. 10.1111/tri.12776 26991858

[18] Steen S Sjoberg T Pierre L Liao Q Eriksson L Algotsson L. Transplantation of Lungs from a Non-heart-beating Donor. Lancet (2001) 357(9259):825–9. 10.1016/S0140-6736(00)04195-7 11265950

[19] Cypel M Levvey B Van Raemdonck D Erasmus M Dark J Love R International Society for Heart and Lung Transplantation Donation after Circulatory Death Registry Report. J Heart Lung Transpl (2015) 34(10):1278–82. 10.1016/j.healun.2015.08.015 26454741

[20] Levvey BJ Westall GP Kotsimbos T Williams TJ Snell GI. Definitions of Warm Ischemic Time when Using Controlled Donation after Cardiac Death Lung Donors. Transplantation (2008) 86(12):1702–6. 10.1097/TP.0b013e3181901f24 19104408

[21] Oto T Levvey B McEgan R Davies A Pilcher D Williams T A Practical Approach to Clinical Lung Transplantation from a Maastricht Category III Donor with Cardiac Death. J Heart Lung Transpl (2007) 26(2):196–9. 10.1016/j.healun.2006.11.599 17258156

[22] Levvey B Keshavjee S Cypel M Robinson A Erasmus M Glanville A Influence of Lung Donor Agonal and Warm Ischemic Times on Early Mortality: Analyses from the ISHLT DCD Lung Transplant Registry. J Heart Lung Transpl (2019) 38(1):26–34. 10.1016/j.healun.2018.08.006 30297241

[23] Reeb J Keshavjee S Cypel M. Successful Lung Transplantation from a Donation after Cardiocirculatory Death Donor Taking More Than 120 Minutes to Cardiac Arrest after Withdrawal of Life Support Therapies. J Heart Lung Transpl (2016) 35(2):258–9. 10.1016/j.healun.2015.10.010 26525404

[24] Lomero M Gardiner D Coll E Haase-Kromwijk B Procaccio F Immer F Donation after Circulatory Death Today: An Updated Overview of the European Landscape. Transpl Int (2020) 33(1):76–88. 10.1111/tri.13506 31482628

[25] Bernat JL D'Alessandro AM Port FK Bleck TP Heard SO Medina J Report of a National Conference on Donation after Cardiac Death. Am J Transpl (2006) 6(2):281–91. 10.1111/j.1600-6143.2005.01194.x 16426312

[26] Lewis J Peltier J Nelson H Snyder W Schneider K Steinberger D Development of the University of Wisconsin Donation after Cardiac Death Evaluation Tool. Prog Transpl (2003) 13(4):265–73. 10.7182/prtr.13.4.w48g8051530058q3 14765718

[27] Wind J Snoeijs MG Brugman CA Vervelde J Zwaveling J van Mook WN Prediction of Time of Death after Withdrawal of Life-Sustaining Treatment in Potential Donors after Cardiac Death. Crit Care Med (2012) 40(3):766–9. 10.1097/CCM.0b013e318232e2e7 21983365

[28] de Groot YJ Lingsma HF Bakker J Gommers DA Steyerberg E Kompanje EJ. External Validation of a Prognostic Model Predicting Time of Death after Withdrawal of Life Support in Neurocritical Patients. Crit Care Med (2012) 40(1):233–8. 10.1097/CCM.0b013e31822f0633 21926586

[29] Suntharalingam C Sharples L Dudley C Bradley JA Watson CJ. Time to Cardiac Death after Withdrawal of Life-Sustaining Treatment in Potential Organ Donors. Am J Transpl (2009) 9(9):2157–65. 10.1111/j.1600-6143.2009.02758.x 19681825

[30] Mason DP Brown CR Murthy SC Vakil N Lyon C Budev MM Growing Single-Center Experience with Lung Transplantation Using Donation after Cardiac Death. Ann Thorac Surg (2012) 94(2):406–11. 10.1016/j.athoracsur.2012.03.059 22608715

[31] Van Raemdonck D Ceulemans LJ Neyrinck A Levvey B Snell GI. Donation after Circulatory Death in Lung Transplantation. Thorac Surg Clin (2022) 32(2):153–65. 10.1016/j.thorsurg.2021.11.002 35512934

[32] Minambres E Rubio JJ Coll E Dominguez-Gil B. Donation after Circulatory Death and its Expansion in Spain. Curr Opin Organ Transpl (2018) 23(1):120–9. 10.1097/MOT.0000000000000480 29120882

[33] Lindemann J Dageforde LA Vachharajani N Stahlschmidt E Brockmeier D Wellen JR Cost Evaluation of a Donation after Cardiac Death Program: How Cost Per Organ Compares to Other Donor Types. J Am Coll Surg (2018) 226(5):909–16. 10.1016/j.jamcollsurg.2018.02.005 29505825

[34] Wall AE Da Graca B Asrani SK Ruiz R Fernandez H Gupta A A Cost Comparison of Liver Acquisition Fees for Donation after Circulatory Death versus Donation after Brain Death Donors. Liver Transpl (2024). 10.1097/LVT.0000000000000328 38190240

[35] Krutsinger D Reed RM Blevins A Puri V De Oliveira NC Zych B Lung Transplantation from Donation after Cardiocirculatory Death: A Systematic Review and Meta-Analysis. J Heart Lung Transpl (2015) 34(5):675–84. 10.1016/j.healun.2014.11.009 25638297

[36] Van De Wauwer C Verschuuren EA van der Bij W Nossent GD Erasmus ME. The Use of Non-heart-beating Lung Donors Category III Can Increase the Donor Pool. Eur J Cardiothorac Surg (2011) 39(6):e175–80. 10.1016/j.ejcts.2011.01.035 21376619

[37] Zych B Popov AF Amrani M Bahrami T Redmond KC Krueger H Lungs from Donation after Circulatory Death Donors: An Alternative Source to Brain-Dead Donors? Midterm Results at a Single Institution. Eur J Cardiothorac Surg (2012) 42(3):542–9. 10.1093/ejcts/ezs096 22371518

[38] Choi AY Jawitz OK Raman V Mulvihill MS Halpern SE Barac YD Predictors of Nonuse of Donation after Circulatory Death Lung Allografts. J Thorac Cardiovasc Surg (2021) 161(2):458–66.e3. 10.1016/j.jtcvs.2020.04.111 32563573 PMC7647952

[39] Holm AM Courtwright A Olland A Zuckermann A Van Raemdonck D. ISHLT Position Paper on Thoracic Organ Transplantation in Controlled Donation after Circulatory Determination of Death (cDCD). J Heart Lung Transpl (2022) 41(6):671–7. 10.1016/j.healun.2022.03.005 35370034

[40] Epker JL Bakker J Kompanje EJ. The Use of Opioids and Sedatives and Time until Death after Withdrawing Mechanical Ventilation and Vasoactive Drugs in a Dutch Intensive Care Unit. Anesth Analg (2011) 112(3):628–34. 10.1213/ANE.0b013e31820ad4d9 21304154

[41] Oto T Rabinov M Griffiths AP Whitford H Levvey BJ Esmore DS Unexpected Donor Pulmonary Embolism Affects Early Outcomes after Lung Transplantation: A Major Mechanism of Primary Graft Failure? J Thorac Cardiovasc Surg (2005) 130(5):1446. 10.1016/j.jtcvs.2005.07.025 16256801

[42] Sanchez-Hidalgo JM Rodriguez-Ortiz L Arjona-Sanchez A Ayllon-Teran MD Gomez-Luque I Ciria-Bru R Super-Rapid Technique in Donation after Circulatory Death Liver Donors: Advantages and Disadvantages. Transpl Proc (2019) 51(1):25–7. 10.1016/j.transproceed.2018.05.034 30655137

[43] Croome KP Barbas AS Whitson B Zarrinpar A Taner T Lo D American Society of Transplant Surgeons Recommendations on Best Practices in Donation after Circulatory Death Organ Procurement. Am J Transpl (2023) 23(2):171–9. 10.1016/j.ajt.2022.10.009 36695685

[44] Jay CL Lyuksemburg V Ladner DP Wang E Caicedo JC Holl JL Ischemic Cholangiopathy after Controlled Donation after Cardiac Death Liver Transplantation: A Meta-Analysis. Ann Surg (2011) 253(2):259–64. 10.1097/SLA.0b013e318204e658 21245668

[45] Heylen L Jochmans I Samuel U Tieken I Naesens M Pirenne J The Duration of Asystolic Ischemia Determines the Risk of Graft Failure after Circulatory-Dead Donor Kidney Transplantation: A Eurotransplant Cohort Study. Am J Transpl (2018) 18(4):881–9. 10.1111/ajt.14526 28980391

[46] Hessheimer AJ Garcia-Valdecasas JC Fondevila C. Abdominal Regional In-Situ Perfusion in Donation after Circulatory Determination of Death Donors. Curr Opin Organ Transpl (2016) 21(3):322–8. 10.1097/MOT.0000000000000315 27050485

[47] Minambres E Royo-Villanova M Dominguez-Gil B. Normothermic Regional Perfusion Provides a Great Opportunity to Maximize Organ Procurement in Donation after the Circulatory Determination of Death. Crit Care Med (2022) 50(11):1649–53. 10.1097/CCM.0000000000005645 36227033

[48] Smith DE Kon ZN Carillo JA Chen S Gidea CG Piper GL Commentary: The Future Is Now-Heart Donation after Circulatory Death. J Thorac Cardiovasc Surg (2022) 164(2):1342–3. 10.1016/j.jtcvs.2020.03.037 34728084

[49] Joshi Y Scheuer S Chew H Ru Qiu M Soto C Villanueva J Heart Transplantation from DCD Donors in Australia: Lessons Learned from the First 74 Cases. Transplantation (2023) 107(2):361–71. 10.1097/TP.0000000000004294 36044329

[50] Shahrestani S Webster AC Lam VW Yuen L Ryan B Pleass HC Outcomes from Pancreatic Transplantation in Donation after Cardiac Death: A Systematic Review and Meta-Analysis. Transplantation (2017) 101(1):122–30. 10.1097/TP.0000000000001084 26950713

[51] Mora V Ballesteros MA Naranjo S Sanchez L Suberviola B Iturbe D Lung Transplantation from Controlled Donation after Circulatory Death Using Simultaneous Abdominal Normothermic Regional Perfusion: A Single center Experience. Am J Transpl (2022) 22(7):1852–60. 10.1111/ajt.17057 35390225

[52] Minambres E Suberviola B Dominguez-Gil B Rodrigo E Ruiz-San Millan JC Rodriguez-San Juan JC Improving the Outcomes of Organs Obtained from Controlled Donation after Circulatory Death Donors Using Abdominal Normothermic Regional Perfusion. Am J Transpl (2017) 17(8):2165–72. 10.1111/ajt.14214 28141909

[53] Jochmans I Hessheimer AJ Neyrinck AP Paredes D Bellini MI Dark JH Consensus Statement on Normothermic Regional Perfusion in Donation after Circulatory Death: Report from the European Society for Organ Transplantation's Transplant Learning Journey. Transpl Int (2021) 34(11):2019–30. 10.1111/tri.13951 34145644

[54] Perez-Villares JM Rubio JJ Del Rio F Minambres E. Validation of a New Proposal to Avoid Donor Resuscitation in Controlled Donation after Circulatory Death with Normothermic Regional Perfusion. Resuscitation (2017) 117:46–9. 10.1016/j.resuscitation.2017.05.030 28591558

[55] Rojas-Pena A Sall LE Gravel MT Cooley EG Pelletier SJ Bartlett RH Donation after Circulatory Determination of Death: The university of michigan Experience with Extracorporeal Support. Transplantation (2014) 98(3):328–34. 10.1097/TP.0000000000000070 24825520

[56] Watson CJE Hunt F Messer S Currie I Large S Sutherland A *In Situ* Normothermic Perfusion of Livers in Controlled Circulatory Death Donation May Prevent Ischemic Cholangiopathy and Improve Graft Survival. Am J Transpl (2019) 19(6):1745–58. 10.1111/ajt.15241 30589499

[57] Oniscu GC Randle LV Muiesan P Butler AJ Currie IS Perera MT *In Situ* Normothermic Regional Perfusion for Controlled Donation after Circulatory Death--The United Kingdom Experience. Am J Transpl (2014) 14(12):2846–54. 10.1111/ajt.12927 25283987

[58] Oniscu GC Siddique A Dark J. Dual Temperature Multi-Organ Recovery from a Maastricht Category III Donor after Circulatory Death. Am J Transpl (2014) 14(9):2181–6. 10.1111/ajt.12808 25056864

[59] Messer SJ Axell RG Colah S White PA Ryan M Page AA Functional Assessment and Transplantation of the Donor Heart after Circulatory Death. J Heart Lung Transpl (2016) 35(12):1443–52. 10.1016/j.healun.2016.07.004 27916176

[60] Zhou AL Ruck JM Casillan AJ Larson EL Shou BL Karius AK Early United States Experience with Lung Donation after Circulatory Death Using Thoracoabdominal Normothermic Regional Perfusion. J Heart Lung Transpl (2023) 42(6):693–6. 10.1016/j.healun.2023.03.001 PMC10192114 36990867

[61] Messer S Large S. Resuscitating Heart Transplantation: The Donation after Circulatory Determined Death Donor. Eur J Cardiothorac Surg (2016) 49(1):1–4. 10.1093/ejcts/ezv357 26487100

[62] Manara A Shemie SD Large S Healey A Baker A Badiwala M Maintaining the Permanence Principle for Death during *In Situ* Normothermic Regional Perfusion for Donation after Circulatory Death Organ Recovery: A United Kingdom and Canadian Proposal. Am J Transpl (2020) 20(8):2017–25. 10.1111/ajt.15775 PMC7540256 31922653

[63] Royo-Villanova M Minambres E Sanchez JM Torres E Manso C Ballesteros MA Maintaining the Permanence Principle of Death during Normothermic Regional Perfusion in Controlled Donation after the Circulatory Determination of Death: Results of a Prospective Clinical Study. Am J Transpl (2024) 24(2):213–21. 10.1016/j.ajt.2023.09.008 37739346

[64] Minambres E Ruiz P Ballesteros MA Alvarez C Cifrian JM Atutxa L Combined Lung and Liver Procurement in Controlled Donation after Circulatory Death Using Normothermic Abdominal Perfusion. Initial Experience in Two Spanish Centers. Am J Transpl (2020) 20(1):231–40. 10.1111/ajt.15520 31265753

[65] Dominguez-Gil B Ascher N Capron AM Gardiner D Manara AR Bernat JL Expanding Controlled Donation after the Circulatory Determination of Death: Statement from an International Collaborative. Intensive Care Med (2021) 47(3):265–81. 10.1007/s00134-020-06341-7 33635355 PMC7907666

[66] Ont.es. Actividad de donaciÃ³n y trasplante â€ OrganizaciÃ³n Nacional de Trasplantes (2024). Available from: https://www.ont.es/https-www-ont-es-informacion-a-los-profesionales-4-actividad-de-donacion-y-trasplante-4-5/ (Accessed January 13, 2024).

[67] Tanaka S Luis Campo-Canaveral de la Cruz J Crowley Carrasco S Romero Roman A Hoyos Mejia L Manuel NaranjoGomez J Effect on the Donor Lungs of Using Abdominal Normothermic Regional Perfusion in Controlled Donation after Circulatory Death. Eur J Cardiothorac Surg (2020):ezaa398. 10.1093/ejcts/ezaa398 33225359

[68] Campo-Canaveral de la Cruz JL Minambres E Coll E Padilla M Antolin GS de la Rosa G Outcomes of Lung and Liver Transplantation after Simultaneous Recovery Using Abdominal Normothermic Regional Perfusion in Donors after the Circulatory Determination of Death versus Donors after Brain Death. Am J Transpl (2023) 23(7):996–1008. 10.1016/j.ajt.2023.04.016 37100392

[69] De Beule J Vandendriessche K Pengel LHM Bellini MI Dark JH Hessheimer AJ A Systematic Review and Meta-Analyses of Regional Perfusion in Donation after Circulatory Death Solid Organ Transplantation. Transpl Int (2021) 34(11):2046–60. 10.1111/tri.14121 34570380

[70] De Wolf J Fadel G Olland A Falcoz PE Mordant P Castier Y Controlled Donation after Circulatory Death Lung Transplantation: Results of the French Protocol Including *In Situ* Abdominal Normothermic Regional Perfusion and *Ex Vivo* Lung Perfusion. J Heart Lung Transpl (2023) 42(8):1093–100. 10.1016/j.healun.2023.03.003 37019731

